# A systematic review and meta-analysis of randomised controlled trials of delayed primary wound closure in contaminated abdominal wounds

**DOI:** 10.1186/1749-7922-9-49

**Published:** 2014-09-06

**Authors:** Boonying Siribumrungwong, Pinit Noorit, Chumpon Wilasrusmee, Ammarin Thakkinstian

**Affiliations:** Section for Clinical Epidemiology and Biostatistics, Faculty of Medicine, Ramathibodi Hospital, Mahidol University, Rama VI Road, Rachatevi, Bangkok, 10400 Thailand; Department of Surgery, Faculty of Medicine, Thammasat University Hospital, Thammasat University (Rangsit Campus), Pathumtani, Thailand; Department of Surgery, Chonburi Hospital, Chonburi, Thailand; Department of Surgery, Faculty of Medicine, Ramathibodi Hospital, Mahidol University, Bangkok, Thailand

**Keywords:** Delayed primary closure, Wound closure, Wound infection, Surgical site infection, Appendicitis, Meta-analysis

## Abstract

A systematic review and meta-analysis was conducted to compare surgical site infection (SSI) between delayed primary (DPC) and primary wound closure (PC) in complicated appendicitis and other contaminated abdominal wounds. Medline and Scopus were searched from their beginning to November 2013 to identify randomised controlled trials (RCTs) comparing SSI and length of stay between DPC and PC. Studies’ selection, data extraction, and risk of bias assessment were done by two independent authors. The risk ratio and unstandardised mean difference were pooled for SSI and length of stay, respectively. Among 8 eligible studies, 5 studies were done in complicated appendicitis, 2 with mixed complicated appendicitis and other types of abdominal operation and 1 with ileostomy closure. Most studies (75%) had high risk of bias in sequence generation and allocation concealment. Among 6 RCTs of complicated appendicitis underwent open appendectomy, the SSI between PC and DPC were not significantly different with a risk ratio of 0.89 (95% CI: 0.46, 1.73). DPC had a significantly 1.6 days (95% CI: 1.41, 1.79) longer length of stay than PC. Our evidence suggested there might be no advantage of DPC over PC in reducing SSI in complicated appendicitis. However, this was based on a small number of studies with low quality. A large scale RCT is further required.

## Introduction

Surgical site infection (SSI) is one of the most common hospital acquired infection [1, 2], which caused by contamination of the wound by exogenous or endogenous bacteria during operations. Once it occurred, patients would suffering from pain, cost of treatments [3, 4], prolonged length of hospital stay, and intangible loss [5].

Delayed primary wound closure (DPC) is a procedure which aims at reducing the rate of SSI by suturing a wound later after proper dressing for 3 to 5 days [6]. The procedure was claimed to decrease bacterial inoculums [7] and increase local wound resistance from increasing wound oxygenation [8] and blood supply [9] from developing granulation tissue. It was firstly applied to traumatic wounds [6] and later was more widely applied to various types of operations (e.g. colonic operations [10, 11], opened tibial fractures [12], gynecologic operations [13]) with demonstration of good efficacy. However, these results were mainly from observational studies that may be prone to selection and confounding biases. In addition, the DPC also has its own disadvantages including pain from routine dressing, necessity for later wound suturing, and increase cost of treatments [14, 15].

The most recent systematic review and meta-analysis comparing the efficacy of DPC by including only randomised controlled trials (RCTs) found no benefit of DPC compared to primary closure (PC) in complicated appendicitis [15]. Since then, more RCTs have been published in which some found benefits of DPC [7, 16] whereas some studies did not [17, 18]. We therefore updated a systematic review and meta-analysis of RCTs which aimed at comparing surgical site infection between DPC and PC in complicated appendicitis underwent open appendectomy and other contaminated abdominal wound.

## Material and methods

### Search strategy

Medline and Scopus databases were used to search relevant studies since initiation to November 2013. Search terms used were (“delayed primary closure” OR “delay primary closure” OR “delayed closure” OR “delay closure” OR “primary closure” OR “wound closure”) AND (“surgical wound infection” [Mesh] OR “superficial surgical site infection” OR “wound infection” OR “superficial SSI”) with limited to randomised controlled trials (RCTs), English, and human for Medline; English, medicine, article, article in press for Scopus. List of references of previous meta-analyses and all eligible studies were also explored for eligibility.

### Studies selection

Two independent authors (B.S. and P.N.) independently selected studies from identified studies using inclusion criteria as follows: study design was RCT, had the outcome of interest as SSI, and had intervention groups as PC and DPC in open surgery. The studies were excluded if they had insufficient data for pooling. If disagreement between the two reviewers occurred, consensus was held with a third party (A.T.) for adjudication.

### Data extraction

B.S. and P.N. extracted data using a standardized data extraction form. Corresponding authors of eligible studies were contacted twice to provide additional data if reported summary data were incomplete. Data from the two reviewers were validated and disagreement was solved by consensus with a third party (A.T.).

### Risk of bias assessment

Risk of bias assessment were done by B.S. and C.W. using the Cochrane tool [19], which consisted of six domains including sequence generation, allocation concealment, blinding, incomplete outcome data, selective outcome report, and other sources of bias. Each item was graded as low or high risk of bias if there was sufficient information to assess, otherwise it was graded as unclear.

### Interventions

The DPC and PC were defined accordingly to individual studies. Briefly, the DPC was defined as a wound that was initially left opened after operation with planning to suture about day 5–7 afterward. The PC was defined as a wound that was sutured immediately after completion of the operation. Wounds that were left open by secondary intention were not considered as DPC and were not included in this analysis.

### Outcomes

The primary outcome was SSI, which was defined according to their original studies. This could be clinical diagnosing using clinical data (e.g., purulent discharge, presence of inflammation) or definite diagnosis proved by specimen culture. Failure to suture as planned in the DPC was also considered as SSI in our analysis. The secondary outcome was length of hospital stay, which was the duration between admission and discharge dates.

### Statistical analysis

A risk ratio (RR) and 95% confidence interval (CI) of SSI between PC and DPC were estimated and pooled using inverse variance method. If heterogeneity of intervention effect was present, the Der-Simonian and Laid method was used for pooling. For length of stay, a mean difference between PC vs DPC was estimated for each study. Data were then pooled using unstandardised mean differences using Der-Simonian and Laid random effect model if heterogeneity was present; otherwise the fixed-effect model was used. If the study did not report mean and standard deviation (SD), these parameters were estimated from median and range in the study using method described by Hozo et al. [20].

Heterogeneity of the studies was assessed using Cochran Q test and a degree of heterogeneity was quantified using I^2^. If either I^2^ ≥ 25% or the Q test was significant, the intervention effects were considered heterogeneous. A meta-regression was performed by fitting co-variables (i.e. age group, type of patients, and use of perioperative antibiotics) into a model to explore sources of heterogeneity. A subgroup or sensitivity analysis was done accordingly if a source of heterogeneity was suggested.

The Egger test and a funnel plot were performed to assess publication bias [21, 22]. If publication bias was suspected either by Egger test or a funnel plot, a contour enhanced-funnel plot and meta-trim and fill were applied where appropriated. Analyses were done using STATA version 12.0. A p value of less than 0.05 was considered statistically significant, except for heterogeneity where 0.10 was used.

## Results

**Figure 1:**
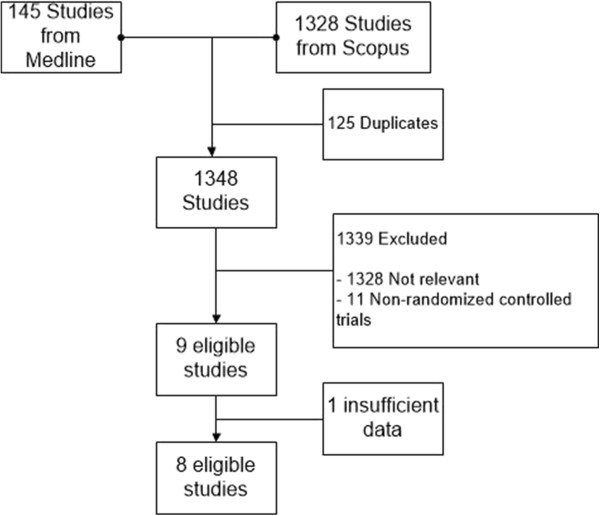
**Studies selection flow.**

A total of 1348 studies (145 and 1328 studies from Medline and Scopus, respectively) were identified after removing duplicates. Screening titles and abstracts were performed and removed 1317 non-relevant studies with reason described in Figure 1, leaving 9 eligible studies to review [7, 16–18, 23–27] (see Figure 1). One study [27] had insufficient data and thus was later excluded after attempting to contact the author twice; leaving 8 studies included in further poolings.

Characteristics of these 8 eligible studies have been demonstrated in Table 1. Most (5/8) RCTs had studied in patients with complicated appendicitis [16, 18, 23–25], 2 studied in mixed complicated appendicitis and other type of contaminated abdominal diseases (e.g. typhoid perforation, traumatic bowel injury) [7, 26], and 1 RCT with ileostomy closure [17]. Studied patients were adults or mixed of adults and children in most studies (6/8) whereas only 2 studies were in children. All studies had performed open surgeries, 5/8 had prescribed prophylaxis antibiotics.

**Table 1:** **Characteristics of eligible studies**

Study	Diseases	Age group	Incision	Prophylaxis antibiotics	Follow up time		Intervention
**Pettigrew 1981** [24]	Perforated and gangrenous appendicitis	Adults and children	Abdominal right lower quadrant (grid iron) and paramedian	No	4 weeks	PC (n = 80)	Interrupted nylon sutures (with topical ampicillin in group B (n = 39)
						DPC (n =42)	Dressing changed was not specified. Wound was closed by interrupted nylon sutures on postoperative day 5
**Tsang 1992** [23]	Perforated and gangrenous appendicitis	Children	Abdominal right lower quadrant	Yes	Not stated	PC (n = 38)	Interrupted nylon sutures
						DPC (n = 25)	Saline dressing daily until day 4 then closed the wound with Steri-Strip
**Cohn 2001** [26]	Perforated appendicitis, other perforated viscus, traumatic injuries more than 4 hours old, or intra-abdominal abscesses	Adults	Abdominal right lower quadrant and midline	Not stated	1 month	PC (n =23)	Wound were closed with skin staples
						DPC (n =26)	Wound packed with saline-soaked gauze, evaluated 3 days after surgery for closure with adhesive strip the next day if appropriate
**Chatwiriya-charoen 2002** [25]	Perforated appendicitis	Children	Abdominal right lower quadrant	Yes	5-14 days after discharge	PC (n =22)	Not stated
						DPC (n =22)	Dressing daily and packed with Betadine gauze 5–10 days until suitable for suture
**Lahat 2005** [17]	Ileostomy closure	Adults	Ileostomy wound	Yes	2 weeks	PC (n =20)	Skin was closed with skin staples
						DPC (n =20)	Wound packed with saline-soaked gauze and were not manipulated until day 3 for evaluation and closure on day 4 with nylon sutures if appropriate
**Duttaroy 2009** [7]	Peptic perforations, typhoid perforations, appendicular perforation/abscesses, penetrating or blunt abdominal injuries with gastrointestinal perforation, or intraperitoneal abscesses	Adults and children	Abdominal	Yes	4 weeks	PC (n =40)	Interrupted 2–0 polyamide sutures
						DPC (n =37)	Packed with saline-soaked gauze for 48 hours then the wound was evaluated for suturing next day with interrupted 2–0 polyamide sutures
**Chiang 2012** [16]	Perforated appendicitis	Adults and children	Right lower quadrant	Yes	Not stated	PC (n =36)	Interrupted nylon sutures
						DPC (n =34)	Packed with Betadine-soaked gauze and changed daily until day 5 or later for DPC
**Khan 2012** [18]	Complicated appendicitis (grossly inflamed, gangrenous, or perforated appendicitis)	Adults	Right lower quadrant	Yes	Not stated	PC (n =50)	Not stated
						DPC (n =50)	Daily or twice dressing until postoperative day 3-5

Risk of bias assessment has been demonstrated in Table 2. All studies had low risk of bias in selective outcome reports and incomplete outcome data. However, 75% of studies had high risk of bias in domains of sequence generations and allocation concealments. None of the studies had blinded assessors because these were surgical techniques.

**Table 2:** **Risk bias assessment of eligible studies**

Author	Domains					
	Sequence generation	Allocation concealment	Blinding	Incomplete outcome data	Selective outcome report	Others sources of bias
**Pettigrew** [24]	Yes	Yes	No	Yes	Yes	No*
**Tsang** [23]	No	No	No	Yes	Yes	Yes
**Cohn** [26]	Unclear	Unclear	No	Yes	Yes	Yes
**Chatwiriya-charoen[** [25]	No	No	No	Yes	Yes	Yes
**Lahat** [17]	No	No	No	Yes	Yes	Yes
**Duttaroy** [7]	Unclear	Yes	No	Yes	Yes	Yes
**Chiang** [16]	No	No	No	Yes	Yes	Yes
**Khan** [18]	Yes	Unclear	No	Yes	Yes	Yes

Yes = Low risk of bias.

No = High risk of bias.

Unclear = Uncertain risk of bias.

*Unbalanced in gangrenous appendicitis between comparison arms.

### Superficial surgical site infection

Five RCTs had compared SSI between PC and DPC in complicated appendicitis. Although the study by Cohn et al. [26] had mixed type of operation, authors reported data for appendectomy separately. This study was therefore included in the main pooling of 6 RCTs (n = 234 vs 182).

**Figure 2:**
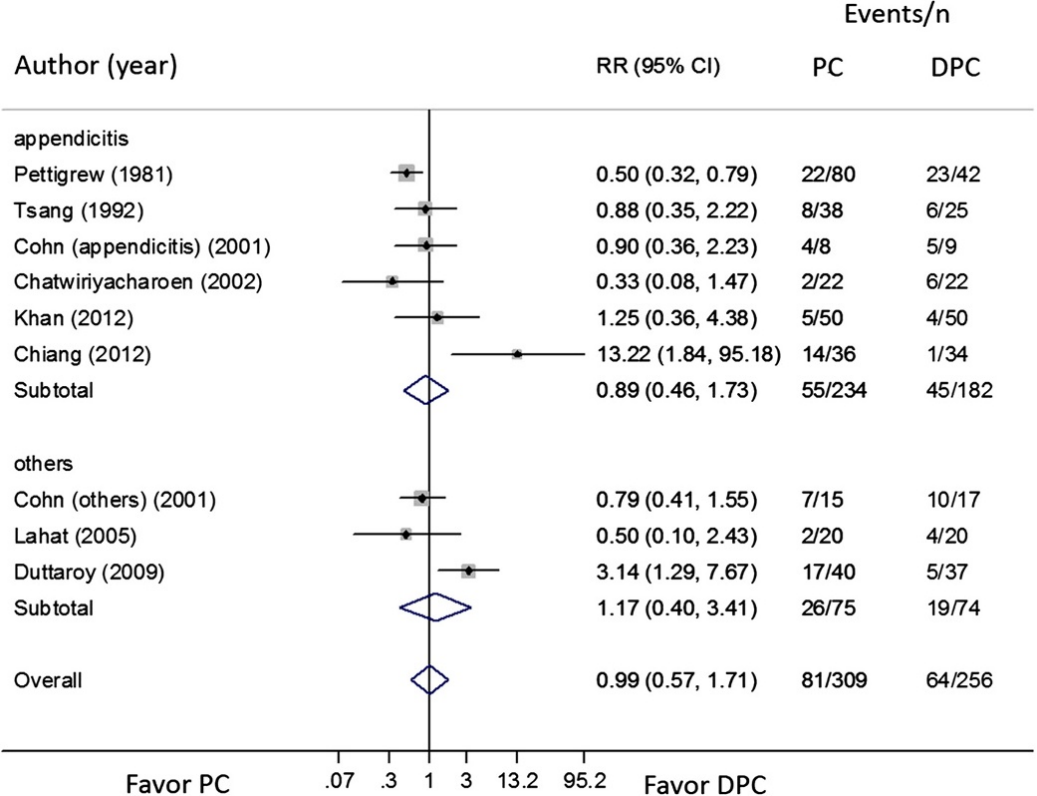
**Forest plot of superficial surgical site infection between primary and delayed primary wound closure according to type of patients.** CI, confidence interval; DPC, delayed primary closure; RR, risk ratio.

The SSI between PC and DPC were highly heterogeneous across 6 RCTs [16, 18, 23–26]. with complicated appendicitis in open appendectomy (Q = 12.87, *p =* 0.025, d.f. = 5, I^2^ = 61.2%) with the incidence of 0.23 (55/234; 95% CI: 0.12, 0.33) and 0.26 (45/182; 95% CI: 0.10, 0.42) in PC and DPC, respectively. The pooled risk RR was 0.89 (95% CI: 0.46, 1.73), demonstrated that the risk of SSI between the closure types were not statistically different, see Figure 2.

Heterogeneity sources were explored by fitting type of studied patients (children [23, 25], adult [18, 26], and mixed children and adults [16, 24]), and use of prophylaxis antibiotics (use [16, 18, 23, 25], not use/not mentioned [24, 26]). None of these sources was identified. A sensitivity analysis was done by including studies with other type of contaminated abdominal wound [7, 17, 26]), yielding then overall pooled RR of 0.99 (95% CI: 0.57, 1.71) with high heterogeneity (Q = 23.20, *p* = 0.003, d.f. = 8, I^2^ = 65.5%), see Figure 2.

**Figure 3:**
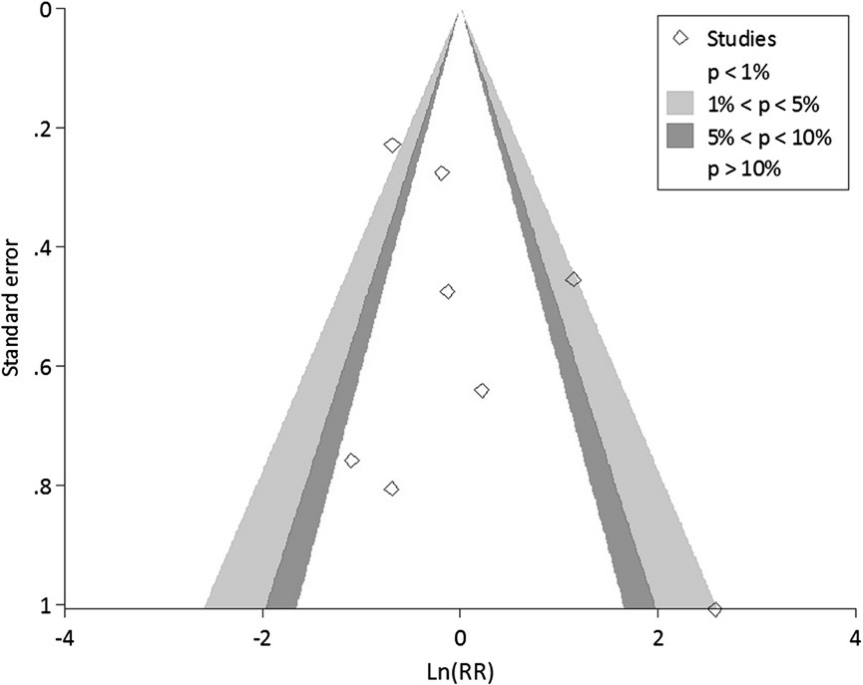
**Contour enhanced funel plots of surgical site infection between primary and delayed primary wound closure.**

Neither the Egger test (Coefficient = 2.17, SE = 1.13, *p* = 0.128) nor the contour-enhanced funnel plot suggested evidence of publication bias for the main pooling RR in appendicitis, see Figure 3.

### Length of stay

**Figure 4:**
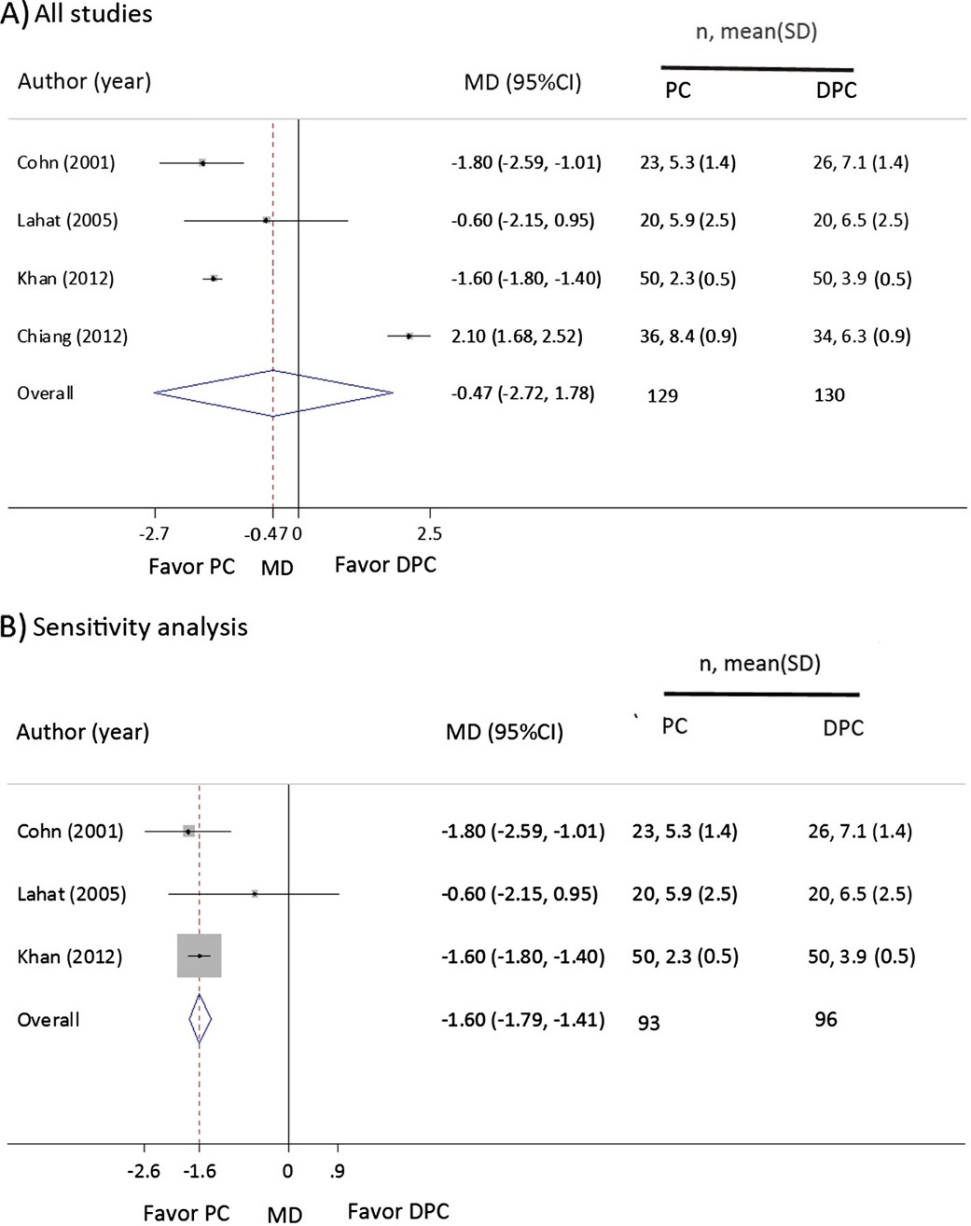
**Forest plot of length of stay after primary and delayed primary wound closure.** CI, confidence interval; DPC, delayed primary closure; MD, mean difference; PC, primary closure; SD, standard deviation, **A)** Pooling overall studies; **B)** Sensitivity analysis by exclude Chiang [16].

There were 4 studies [16–18, 26] which compared length of stay between PC and DPC with sample sizes of 129 and 130 patients, respectively. The length of stay was non-significantly different between PC and DPC with the pooled mean difference of -0.5 day (95% CI: -2.7, 1.8), see Figure 4. However, the length of stays were highly heterogeneous (Cochran Q of 247.64, d.f. = 3, *p* < 0.001 and I^2^ of 98.8%), and the forest plot suggested that the study from Chiang et al. [16] was far different from the others due to the number of readmission days was accumulated in the total length of stays in the PC group whereas other studies accounted this only one episode of admission. Therefore, sensitivity analysis was done by excluding this study which yielded significantly shorter hospital stays in PC than in DPC with the pooled mean difference of -1.6 days (95% CI: -1.8, -1.4) with I^2^ of 0%. This demonstrated that PC had significantly 2 days shorter length of hospital stay when compared to DPC. No publication bias was suggested by Egger test (*p* = 0.685) and contour-enhanced funnel plot.

## Discussion

We performed a systematic review and meta-analysis to assess efficacy of PC and DPC considering only RCTs in contaminated abdominal wound, which mainly focused on complicated appendicitis with open appendectomy. Our results suggested that the SSI rates were not significantly different between the two techniques in either open appendectomy or other operations. In addition, the length of hospital stay was 2 days significantly longer in DPC than PC. Our finding was consistent with a previous systematic review and meta-analysis that found lack of benefit of DPC over the PC in complicated appendicitis in children [15]. However, our results were pooled based on high heterogeneity of effects without explanation of source of heterogeneities.

Our study focused on studies applying only open appendectomy. In the current era with increasing use of minimally invasive approach, evidences from observational studies showed that laparoscopic appendectomy was better than open appendectomy in decreasing SSI rate in complicated appendicitis [28, 29], but conversion rate from laparoscopic to open appendectomy was as high as 13% to 16% [29, 30]. Although the laparoscopic appendectomy has advantages over the conventional open appendectomy, this approach is mostly available in tertiary cares or school of medicine hospitals, and it also very much depends on experience of surgeon. Therefore, open appendectomy is still useful where limited resources.

Contamination of the wound from environmental bacteria during dressing can increase the risk of infection in DPC [7]. Therefore, frequency of dressing, sterile technique, and suturing should be considered and concerned before applying DPC in a different setting.

The SSI after DPC can be classified into two types, i.e., failure to close and after resuture the wound. The former causes less morbidity than the later because of pain, discomfort, and suffering of SSI during infection time before diagnosis is made. Although our results found similar SSI after PC and DPC, applying PC should be cautioned particularly in highly contaminated wounds or in immune-compromised hosts. Risk classification scores that can predict SSI after PC and after resuturing should be able to aid physicians to make decisions which technique between DPC and PC should be applied.

The strength of our studies is that we included only RCTs that could minimize selection and confounding biases. A sensitivity was performed by including RCTs with other operations in the main pooling of RCTs with complicated appendectomy. A pooled magnitude of effect of DPC vs PC was estimated and reported. However, our results were pooled based on high heterogeneity across included studies. A number of included RCTs was also quite small. As a result, the range of estimation of effect was imprecise, i.e., varied from 0.46, 1.73. Furthermore, most studies (75%) had high risk of bias in sequence generation and allocation concealment. Therefore, further large scale RCTs or updated meta-analysis is required to confirm our results.

## Conclusion

DPC had no advantages over PC to reduce the rate of SSI with longer hospital stay in complicated appendicitis. However, applying PC in patients with high risk of SSI should be cautioned.

## Abbreviations

CI: Confidence interval
Df: Degree of freedom
DPC: Delayed primary wound closure
PC: Primary wound closure
RR: Risk ratio
SSI: Surgical site infection.
